# Climate distress and social identity: bringing theory to clinical practice

**DOI:** 10.3389/fpsyg.2023.1126922

**Published:** 2023-08-31

**Authors:** Marc O. Williams

**Affiliations:** South Wales Doctoral Programme in Clinical Psychology, Cardiff University, Cardiff, United Kingdom

**Keywords:** climate distress, climate anxiety, collective emotions, social isolation, hope, pro-environmental behavior, social identity approach, clinical practice

## Abstract

Guidance for supporting individuals with climate distress often lacks a theoretical foundation to account for its social dimension. This paper argues for the value of the social identity approach (SIA) for understanding and supporting individuals with climate distress in clinic. Three aspects of climate distress are considered: social isolation, collective emotions, and climate action. It is posited that the SIA can guide interventions in a way that is tailored to the specific social dynamics entailed in each client’s climate distress. The paper also considers how clinicians can weigh up the potential advantages and disadvantages of interventions that are commonly advised for these individuals, such as contact with nature and engaging in collective action. The author is a clinical psychologist and lecturer researching climate distress.

## 1. Introduction

Climate distress encapsulates an array emotional responses to human-caused environmental change, including anxiety (Clayton, 2020), depression and anger (Stanley et al., 2021), and grief (Cunsolo and Ellis, 2018). Climate distress is experienced within enveloping layers of social, political, and ecological realities, whose influence ripples inward to the individual (Crandon et al., 2022). A global survey of young people highlighted that individual and societal responses to climate change are woven tightly together: climate anxiety was correlated with feelings of government betrayal, and 48% of the sample reported that others had ignored or dismissed their attempts to discuss climate change (Hickman et al., 2021). Clayton and Karazsia (2020) developed a measure of climate anxiety with items that emphasize its social aspects, such as: “My friends say I think about climate change too much” (p.4). A large majority of those reporting climate distress in Tuvalu described impaired functioning as a result, including avoiding social activities and going out (Gibson et al., 2020). Practitioners have begun to see individuals presenting with climate distress in clinic (e.g., Seaman, 2016), and there is a duty to understand the social dynamics entailed and how psychological interventions might address them.

Reviewed existing guidance for supporting the social aspects of climate distress. Recommendations included discussing societal responses to climate change in therapy, such as collective guilt (Randall, 2005; Lewis, 2018), and promoting group involvement to enhance social connection and collective action against climate change (Conyer, 2019). However, the review also revealed that most guidance for climate distress does not explicitly draw on a particular theoretical or therapeutic approach; without a coherent theoretical understanding of climate distress and its social aspects, there can be no systematic approach to helping individuals in a way that is beneficial, replicable, and that can be delivered at-scale.

Social psychology, which concerns itself with people in their interface with society (Tesser and Schwarz, 2008), is well-positioned to account for some of the unique aspects of climate distress, such as collective guilt. It also harbors the potential to guide the development of interventions that can simultaneously mitigate climate distress and promote collective environmental action. In this paper, I argue that the social identity approach (SIA) is a particularly relevant body of theoretical work for understanding the social dimension of climate distress. The SIA encompasses insights from two theoretical accounts of social cognition and behavior: social identity theory (Tajfel and Turner, 1979) and self-categorization theory (Turner et al., 1987) (e.g., Tanis and Postmes, 2005). Due to the specific focus of this paper, a comprehensive review of the two theories comprising SIA surpasses its remit. In general, the SIA postulates that humans shift between an individual- and a group-based way of identifying, depending on context; the activation of a social category in any given moment can lead an individual to “depersonalize,” in which they perceive themselves primarily as a representative of a particular in-group (self-categorization theory; Turner et al., 1994). There are various ramifications of defining oneself at a member of a collective, including the motivation to see one’s in-group positively on valued dimensions in relation to out-groups and trying to protect the interests of the in-group (social identity theory; Tajfel and Turner, 1979).

This paper relates insights from the SIA to three aspects of climate distress: social isolation, collective emotions, and climate action. I will suggest ways in which the SIA could be of use therapeutically in this context for reducing isolation, promoting collective hope, and encouraging pro-environmental action in a way that has a net benefit for well-being. In so doing I aim to demonstrate the relevance of SIA for guiding therapeutic research and practice in relation to climate distress.

## 2. From isolation to integration: the role of collective identity

The SIA predicts that individuals who perceive a threat to the interests of their in-group will be more concerned about climate change, and the evidence supports this (e.g., younger groups tend to show higher climate concern) (Mackay et al., 2021). Climate distress is associated with social emotions such as mistrust, a sense of betrayal by governments, and indignation toward others (Hickman et al., 2021; Jovarauskaite and Böhm, 2021). Young adults have reported feeling anger and betrayal in relation to having been abandoned by older adults in relation to climate change (Jones and Davison, 2021). Climate distress can be associated with social withdrawal, which potentially exacerbates loneliness and mistrust (Budziszewska and Jonsson, 2021; Hajek and König, 2022). Societal stigma might also contribute to climate distress; there is evidence that being concerned about the environment can attract disapproval from others (MacInnis and Hodson, 2017), which could make it harder for those with climate distress to maintain a positive social identity. Isolation of those with climate distress might maintain itself by stymying communication with others, thereby entrenching ideological disparities (Jost et al., 2022). Society is less likely to develop an adequate response to the climate crisis if lines of communication with those experiencing climate distress are frayed.

Haslam et al. (2016) have argued that an effective therapeutic intervention for social isolation and withdrawal should be grounded in theories of social process. These authors reported a comprehensive group programme founded on the SIA for optimizing mental health by harnessing the power of social group memberships. The programme involves participants in social identity mapping: a process of identifying one’s various social groups, subjective belongingness to each group, as well as each group’s personal importance (Cruwys et al., 2016). Participants are then guided through a process of strengthening existing social connections and envisioning new social connections that they could build (Haslam et al., 2016). The authors reported that their sample of socially-isolated young adults showed improvements on a range of mental health outcomes that was predicted by an increase in the number of perceived social group memberships.

Preliminary data suggest that having numerous social ties can enhance resilience in the face of climate change: better psychological resilience and lower distress following the Australian wildfires of 2019–2020 were predicted by having robust social connections pre-wildfire (Cruwys et al., 2023). While existing guidance for climate distress highlights the role of engagement with others to enhance social connection (Conyer, 2019), clinicians can finesse these approaches for each individual by formulating the specific social-cognitive processes maintaining *their* climate distress. For individuals who feel alone and isolated, feel connected to few social groups, or who experience stigma tied to a negative social identity, a therapeutic intervention based on the SIA could alleviate climate distress by encouraging contact and camaraderie with others who share their concerns. In practice, professionals could explore with their clients the benefits joining climate cafés, where feelings about climate change are discussed in a non-judgmental and supportive environment with others who are concerned about the climate (Justes et al., 2017). Clients could also be helped to consider joining environmentalist groups, where interaction with others from a range of social groups (e.g., people from different generations) could help rebuild trust by enhancing the number of perceived group memberships.

A more comprehensive perspective on this issue can be achieved by considering nature-based identity: connectedness to nature is a sense of oneness with the natural world (Mayer and Frantz, 2004) and has been conceptualized as a form of collective identity with nature (Schmitt et al., 2019). Meta-analyses have shown strong evidence for the association between higher levels of connectedness to nature and higher well-being and happiness, and there is evidence that nearby nature buffers against social isolation (Capaldi et al., 2014; Cartwright et al., 2018; Pritchard et al., 2020). However, nature connectedness might pose risks to mental well-being in a climate change context. A study of Brisbane inhabitants found higher levels of nature connectedness to be associated with more psychological distress, and the authors surmised that recent floods and relaxed environmental laws might have been more concerning to those who feel more connected to nature (Dean et al., 2018). Another study in Australia, which collected survey data following a severe bushfire, reported similar findings (Curll et al., 2022). In a United Kingdom population sample, those who identified more strongly with nature showed higher levels of cognitive-emotional and functional impairment relating to climate distress (Whitmarsh et al., 2022). These observations can be understood through the lens of self-categorization theory, in which distress arises from perceived injustice to the in-group (nature) at the hands of an out-group (Smith, 1993; Turner et al., 1994).

The complex relationship between nature connectedness and mental health has yet to be incorporated into guidance for supporting individuals with climate distress. Noted that connecting people with nature is common advice for helping people with climate distress; however, there is an ethical dilemma of enhancing connectedness to nature during the Anthropocene (Larson et al., 2022), and psychological therapies that employ inappropriate techniques can be harmful (Parry et al., 2016). Guidance for working with climate distress should therefore take in both the potential benefits and risks of doing so. For those who identify strongly with nature, a more guided approach to contact with nature and a space to explore and make sense of one’s feelings would be more appropriate than “prescribing” nature contact that clients undertake without such guidance. Ecotherapy is an approach that brings consciousness to one’s nature contact, and provides a space for clients to explore their reactions to the natural world and their identity in relation to it (Doherty, 2016). A mindful stance is often encouraged in ecotherapy (Doherty, 2016), and by bringing intentional, non-judgmental awareness to the present moment, one can increase nature connectedness (Sheffield et al., 2022) whilst also maintaining a psychological distance from painful thoughts (“cognitive defusion”) (Masuda et al., 2004; Blackledge, 2007). Having examined how to reduce isolation for those with climate distress, I now turn to consider how social group identification can cultivate an array of collective emotions and their role in climate distress.

## 3. Harnessing hope: the role of collective emotions

Collective identity gives rise to collective emotions, i.e., emotions that arise from group membership (Smith and Mackie, 2015). Emotional responses to others’ behavior is often a feature of climate distress, such as a sense of collective guilt. While emotions such as collective guilt can increase engagement with climate action (Ferguson and Branscombe, 2010), individuals may unjustly shoulder an excessive burden of guilt for others’ actions.

Existing guidance suggests that individuals can be reassured that they are accepting an unreasonable amount of responsibility. While this approach could alleviate feelings of excessive guilt, understanding the power of a *shared* responsibility for mitigating climate change might open the door to another emotion: hope. There can be many kinds of hope, and practitioners would benefit from understanding which kinds are most beneficial to well-being. Ojala (2012) created a measure of “constructive hope,” comprising trust in other actors, trust in laypeople’s efforts, and positive re-appraisal (e.g., putting things in a historical perspective by considering the increase in societal awareness of climate change); constructive hope was found to be uniquely predictive of pro-environmental behaviors in Swedish teenagers, whereas hope based on denial was not. Similarly, in a United States population survey, hopefulness in the context of climate change was found to be most common among those who cited seeing or learning about others’ actions to address climate change (Marlon et al., 2019). Viewed from an SIA perspective, the kind of hope described in these studies appears to be a collective emotion: hope that humans as a group are sufficiently aware of the problem and able to act effectively.

Practitioners may be able to minimize excessive guilt and foster hope by helping clients consider their place within a collective. Cultivating collective hope could be achieved by encouraging clients to consider past successes in social cooperation to tackle global problems and the increasing recognition of climate change worldwide. Drawing on these examples might help clients to perceive their collective potential to affect meaningful change. While hope by itself is insufficient for tackling climate change, in the next section I will explore how collective emotions can fuel collective climate action in a way that also mitigates climate distress.

## 4. Action amplified: the therapeutic value of working as a collective

According to Social Identity Model of Pro-Environmental Action (SIMPEA), collective emotions, such as guilt and anger resulting from the awareness of humans’ harmful environmental actions, can motivate action to maintain a positive view of the in-group (Fritsche et al., 2018). Collective guilt has been found to mediate between awareness of global warming and willingness to engage in climate mitigation behaviors (Ferguson and Branscombe, 2010). Other collective emotions, such as anger about human-caused climate change, also appear to predict climate action (Stanley et al., 2021).

Common advice for supporting individuals with climate distress is to encourage pro-environmental behavior in order to bring actions into alignment with environmental values, and collective action is advised in order to gain emotional support from groups and to spur people on to act. This is in line with the call for individuals to engage in climate action; Nielsen et al. (2021) argued that individuals have a variety of roles extending beyond the household (as participants in organizations and workplaces, as consumers, and as citizens who vote in elections) and can have large impacts by influencing policy that will lead to widespread systemic changes. On the other hand, Van Nieuwenhuizen et al. (2021) drew parallels between climate activism and “minority tax” (Miller et al., 2021), in which certain groups (e.g., young people) shoulder the burden for climate action. Indeed, people with higher levels of climate distress are already those who tend to show higher levels of pro-environmental behavior (Whitmarsh et al., 2022). Therefore, while encouraging climate action could be beneficial for individuals with climate distress in some cases, care should be taken not to contribute to a disproportionate expectation being placed on people with climate distress to take action. The Association of Clinical Psychologists (United Kindom) released a position statement that clinical psychologists should consider peaceful participation in protests to highlight the urgency of the climate crisis (Morgan et al., 2020). Other organizations have released similar statements, including the American Psychological Association, which produced a report urging members to take action against climate change (APA, 2022).

While there is evidence that collective action lessens the association between climate anxiety and depression (Schwartz et al., 2022), it also has the potential to engender hopelessness and burnout (Charlson et al., 2022). Furthermore, those perceived as environmental “do-gooders” can be socially sanctioned with ridicule, derogation, and social exclusion (MacInnis and Hodson, 2017; Bolderdijk et al., 2018). Klas et al. (2019) found that environmentalists who engage in collective action, such as protests, attract more negative attitudes than those who engage in private-sphere behavior such as recycling. The SIA can explain these observations: members of groups are easily triggered to demean members of other groups as a means of enhancing their self-esteem (social identity theory; Tajfel and Turner, 1979). Any discussion about engaging in collective action should consider the benefits as well as the potential (social) costs, and should help clients to weigh up options.

Understanding the psychological mechanisms by which working as a collective could be protective against climate distress is important in order for therapists to be tailored in their approach and target the specific mechanisms that maintain climate distress for each individual client. Collective action could achieve some of the outcomes discussed in Section 2: tackling social isolation, building a positive social identity, or extending one’s social group memberships. However, being part of a collective that is taking action could also enhance collective efficacy, i.e., the degree to which a person perceives their group to be effective in achieving its goals (Bandura, 2000). There is evidence, for example, that taking part in collective action builds collective efficacy via a sense of empowerment in challenging the status quo (Drury and Reicher, 2005).

Psychological therapies often focus on building self-efficacy, or the belief in one’s own personal ability to achieve a goal (Padesky and Mooney, 2012). Building self-efficacy in therapy can entail engaging in behaviors to learn about one’s own abilities to deal with a range of situations, and therapists can help clients to be attentive to the many outcomes of their actions so as not to miss or discard outcomes that contradict thier predictions (Beck, 1997). Likewise, for those with low confidence in humans’ ability to address climate change (i.e., low collective efficacy), practitioners could encourage engagement in collective action as well as a careful operationalization of what counts as a “successful” outcome (e.g., media exposure of the group’s actions and the number of others who signed up to the action).

## 5. Conclusion

This paper has proposed the relevance of the SIA for conceptualizing the social dynamics entailed in climate distress and ways of approaching these dynamics in a therapeutic context. I have made suggestions for interventions incorporating insights from SIA that might be helpful for climate distress in a way that mitigates a sense of social disconnection that those with climate distress can experience, relieves excessive guilt, builds hope and collective efficacy, and allows for a considered approach to pro-environmental behavior and contact with nature that minimizes the potential for harm. Therapists should consider how to empower individuals with climate distress to engage with the crisis in a way that is beneficial to their mental health and does not place a disproportionate burden on these individuals; part of this is acknowledging practitioners’ own responsibility to engage with the issue.

## Data availability statement

The original contributions presented in the study are included in the article/supplementary material, further inquiries can be directed to the corresponding author.

## Author contributions

The author confirms being the sole contributor of this work and has approved it for publication.

## Conflict of interest

The author declares that the research was conducted in the absence of any commercial or financial relationships that could be construed as a potential conflict of interest.

## Publisher’s note

All claims expressed in this article are solely those of the authors and do not necessarily represent those of their affiliated organizations, or those of the publisher, the editors and the reviewers. Any product that may be evaluated in this article, or claim that may be made by its manufacturer, is not guaranteed or endorsed by the publisher.
